# Lung Nodule Malignancy Prediction From Longitudinal CT Scans With Siamese Convolutional Attention Networks

**DOI:** 10.1109/OJEMB.2020.3023614

**Published:** 2020-09-11

**Authors:** Benjamin P. Veasey, Justin Broadhead, Michael Dahle, Albert Seow, Amir A. Amini

**Affiliations:** University of Louisville 5170 Louisville KY 40208 USA

**Keywords:** Lung cancer diagnosis, X-ray CT, longitudinal studies, siamese networks, deep learning

## Abstract

*Goal:* We propose a convolutional attention-based network that allows for use of pre-trained 2-D convolutional feature extractors and is extendable to multi-time-point classification in a Siamese structure. *Methods:* Our proposed framework is evaluated for single- and multi-time-point classification to explore the value that temporal information, such as nodule growth, adds to malignancy prediction. *Results:* Our results show that the proposed method outperforms a comparable 3-D network with less than half the parameters on single-time-point classification and further achieves performance gains on multi-time-point classification. *Conclusions:* Attention-based, Siamese 2-D pre-trained CNNs lead to fast training times and are effective for malignancy prediction from single-time-point or multiple-time-point imaging data.

## I. Introduction

Lung cancer is the leading cause of cancer-related death among both men and women, accounting for 18.4% of the total cancer deaths world-wide [1]. Screening high-risk patients with low-dose Computed Tomography (CT) can lead to earlier treatment and increase the survival rate [2]. However, cancer diagnosis remains a challenging problem with this modality due to the subtle visual differences between benign and malignant nodules in CT images. Hence, Computer-aided Diagnosis (CADx) systems may prove useful in assisting radiologists in the malignancy prediction task.

Recent CADx systems for lung nodule classification are dominated by deep learning strategies for feature extraction and classification. 2-D Convolutional Neural Networks (CNNs) [3], [4], multi-view 2-D CNNs [5], and 3-D CNNs [6], [7] are the typical deep learning methodologies that have been applied to the thoracic CT scans.

**Fig. 1.**
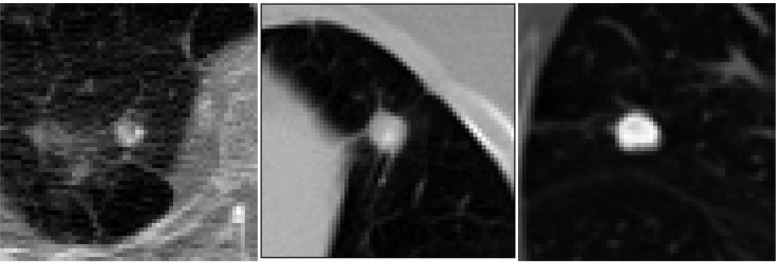
Left to right: central slices of three nodules taken from patient numbers 658, 766, and 974 of the LIDC database. In each case, two radiologists labeled them as likely benign and two as likely malignant. The figure illustrates the inter-variability in radiologists’ diagnoses.

For training and testing, by and large these studies made use of the Lung Image Database Consortium Image Collection (LIDC) [8]. Malignancy of nodules in LIDC, however, is not confirmed with biopsy. Instead, malignancy is visually scored in the range of [1]–[5] with 1 indicating a low probability of malignancy and 5 indicating a high probability of malignancy. The LIDC ground truth for training and testing in prior CADx systems was derived from the median malignancy scores [6] or average of malignancy scores [4], [7] of 3 or 4 radiologists. While plausible, this approach does not consider the variability among different radiologists' diagnoses. To illustrate this point, mid-axial slices of three nodules from the LIDC database are shown in Fig. 1. Nodules shown in this figure belong to patients 658, 766, and 974 of LIDC database. For all nodules in this figure, two radiologists classified them as very suspicious (assigning a malignancy score of 4 or 5) while another two assigned a malignancy score of 1 out of 5 to the same nodules indicating their certainty that the nodules were benign. Such inter-variability reaffirms the previously reported finding that lung cancer is the third most frequently missed diagnosis based on expert readers' visual assessment [9].

The National Lung Screening Trial (NLST) [2] enrolled more than 53,000 high risk subjects between 55 and 74 years of age. The study revealed that participants who received low-dose helical CT scans had a 15 to 20 percent lower risk of death from lung cancer than participants who received standard chest X-rays. The NLST database, which is much larger than the LIDC database, includes scans from multiple time points (typically t_0_, t_1_, and t_2_ scans taken one year apart) as well as biopsy confirmations for suspicious lesions.

In the lung cancer CADx literature to date, few studies have utilized nodules with biopsy confirmed diagnoses. In an early attempt [10], researchers extracted a 219-dimensional feature vector quantizing shape, location, and texture of nodules and tested different classifiers to determine the possibility of predicting cancer from radiomic features. Alternatively, a combination of CNN and radiomic features were unified in [11].

Previously, we described a dataset of challenging, early-stage lung nodules from the NLST database [12] and developed a 2-D convolutional neural network / recurrent neural network (CRN) framework for malignancy prediction. In this paper we improve on our previous results in several ways. First, we develop an attention-based network which outperforms the CRN model while still allowing the use of 2-D feature extractors similar to those developed for the ImageNet challenge. Furthermore, we extend our prior results which only considered single-time-point nodule classification to multiple time points. To do this, we leverage network submodules that share identical architectures and weight parameters and that process input volumes in tandem. This structure is typically referred to as a Siamese-style architecture. The approach provides flexibility by allowing various number of inputs to be processed concurrently while also reducing the number of overall weight parameters since they are shared across twin branches. As we and others have previously shown [12]–[14], 2-D CNN-based systems can effectively encode 3-D information from multiple 2-D slices. This is important because it permits pre-trained 2-D networks with substantially fewer parameters to be applied to 3-D image data with comparable results.

Others have applied attention networks to lung nodules [15]–[17] for malignancy classification. Our method differs from those by focusing on slice-wise attention for reducing network parameters to learn an appropriate nodule malignancy classifier. Our method then uses Siamese-like processing of multiple input volumes to process multiple time points at once to improve classification performance.

The rest of the paper is organized as follows: Section II reviews our dataset and illustrates details of the methodology, Section III reports the experimental results, Section IV discusses the findings, and Section V provides conclusions.

## II. Materials and Methods

An important aspect of this paper is its use of a dataset useful for developing lung nodule malignancy classifiers for longitudinal data. The following sections first discuss the NLSTx dataset previously introduced in [12] in detail before introducing a convolutional attention network (CAN) for classifying 3-D image data which utilizes a 2-D convolutional neural network (CNN) feature extractor with ImageNet weights.

### A. NLSTx Dataset [12]

Data from 15,000 participants in the NLST were obtained from the National Cancer Institute (NCI) - most of these scans are normal and do not contain nodules. In order to select a set of malignant nodules that are early-stage and/or visually challenging to diagnose, we filtered the subjects to include those who had malignant nodules in the first time point, t_0_, but whose scans had not lead to the clinical need for a biopsy which is typically performed for suspicious nodules. We deduced that radiologists did not regard these nodules to be malignant during the initial screening interval but became suspicious after a subsequent scan. For improving prognosis, these are the most important nodules requiring accurate diagnosis. In addition to malignant nodules, the database also includes benign nodules; for these, we selected data from subjects who had only one nodule marked in the NLST clinical notes. This avoided any potential human errors when our radiology collaborators matched clinical notes to image data.

In order to ensure that only truly malignant nodules were selected for NLSTx and that we would only include nodules scanned in multiple intervals, all of the following conditions had to be satisfied: 1) There was a positive reading in either the 2^nd^ or 3^rd^ interval, 2) a positive biopsy result was noted, and 3) anatomical location of the cancer was identified and listed on the NLST Participant Data sheet. Benign nodules then were selected for patients who had three consecutive negative screenings.

**Fig. 2.**
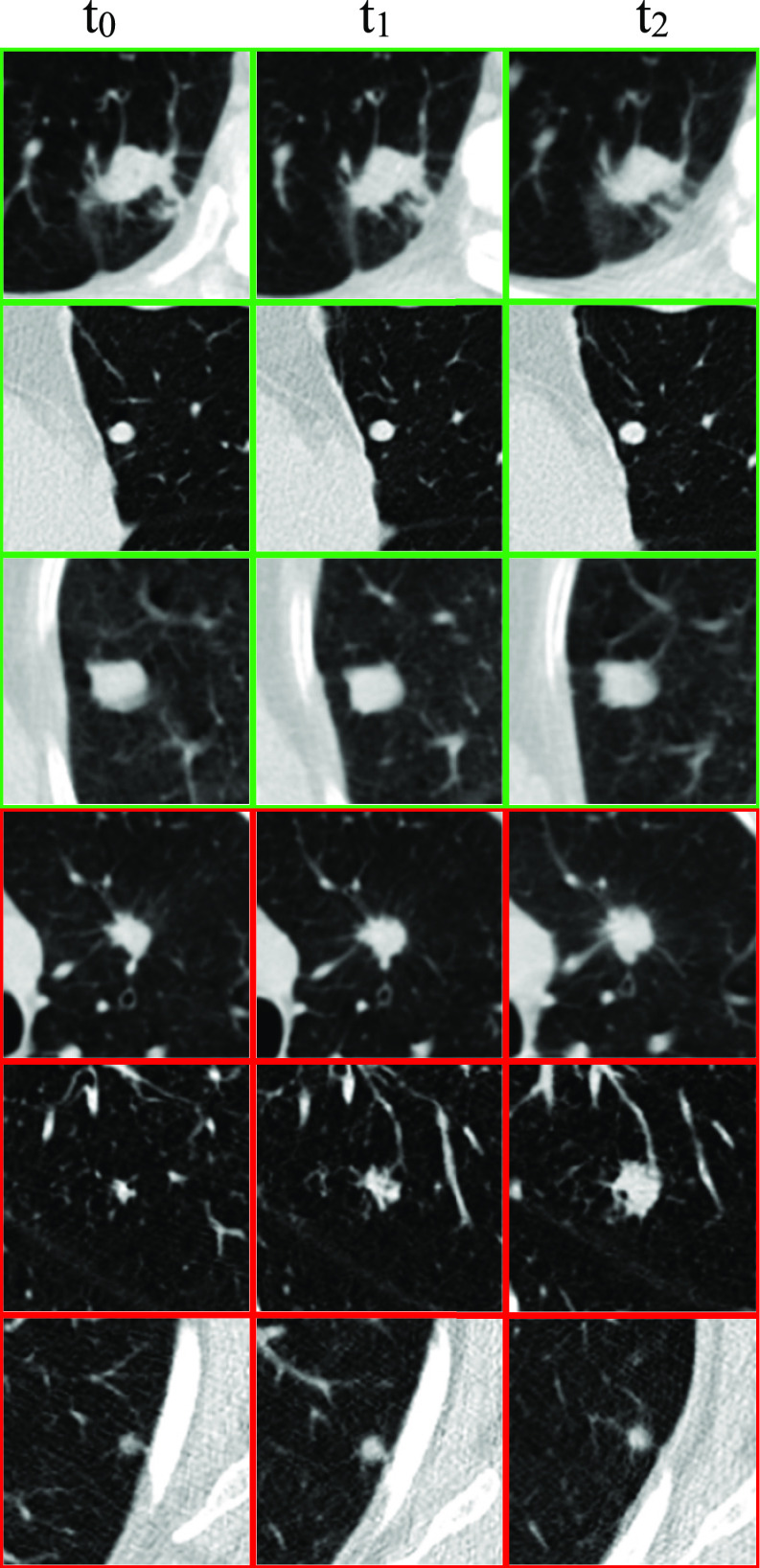
The center slice of three benign (green border) and three malignant (red border) nodules in the NLSTx dataset at three time points (all viewed at 80 mm^3^). Diagnosis in each malignant case was confirmed by biopsy.

Since specific nodule locations are not provided in the NLST dataset, our collaborating radiologists used the anatomical locations provided in the NLST clinical notes to find specific, pixel-level nodule locations through placement of a bounding box on the axial slice with largest nodule diameter. In total, 857 nodules from distinct subjects – 207 malignant and 650 benign – each at multiple time points – were derived from NLST. Subjects in NLSTx who have benign nodules have three scans – one scan taken annually – while subjects with malignant nodules have either two or three scans taken annually. See Fig. 2 for examples of nodules in NLSTx.

Scans selected for the NLSTx dataset have slice thickness ≤ 2.5 mm (mean: 2.32 mm) while in-plane resolution has the range 0.48-0.89 mm (mean: 0.66 mm). Table I gives a statistical overview of the nodule diameters split between benign and malignant classes. Not surprisingly, we notice that the benign nodules stay rather stable in size across scan intervals while the malignant nodules steadily increase in size. In summary, the proposed NLSTx database improves on the widely used LIDC database in that all malignant nodules have independently confirmed biopsies with scans at multiple time points.

**TABLE I:** NLSTx Dataset Statistics

	Benign			Malignant		
Scan Interval	Diameter	Mean	Median	Diameter	Mean	Median
t_0_	4.98-38.67	10.28	9.31	7.03-76.37	17.53	16.29
t_1_	4.32-33.20	10.23	9.30	7.87-97.97	19.28	17.1
t_2_	4.52-34.22	10.4	9.45	8.09-91.59	21.61	19.14

Statistics of nodule diameters in NLSTx (all in mm).

### B. Convolutional Recurrent Network Architecture

In our previous work [12], we developed convolutional recurrent networks (CRNs) for single-time-point malignancy classification. These networks process individual slices of a 3-D input volume and pass the subsequent per-slice features to a recurrent network module. The recurrent module then sequentially compresses the feature series to a reduced form while maintaining semantically relevant features for malignancy classification. A fully-connected layer is then used for the final classification.

Our work in this paper improves on this by replacing the recurrent module with a slice-wise attention mechanism. The attention mechanism is not restricted to sequentially processing information like a recurrent module and, instead, can process each slice's features in parallel. This results in a good balance between CRNs and 3-D CNNs by providing the overall network with a theoretical reduction in inference speed while maintaining a reduced number of network parameters when compared to a 3-D CNN. For these reasons, we will compare CRNs and 3-D CNNs against our proposed approach in this paper.

### C. Convolutional Attention Network Architecture

We propose a parameter-efficient Convolutional Attention-based Neural Network (CAN) that uses a 2-D CNN for extracting in-plane features and a slice-wise attention mechanism for integrating information across multiple slices. The advantage of the proposed approach is that a variety of 2-D networks may be plugged in to derive best performance from the framework. The proposed framework allows us to directly utilize the same 2-D networks that have been developed for the analysis of 2-D natural scene images. In addition, the attention mechanism reduces the size of the feature vector entering the classifications layers. Since most network parameters exist in the fully-connected layers and are dependent on the size of the incoming vectors, this significantly reduces the overall number of network parameters. This is especially the case when compared with an equivalent 3-D network that has no such feature reduction. We posit that this lower number of features eases optimization of the classifier and helps the network achieve higher performance.

#### 1) Single-Time-Point Classification

**Fig. 3.**
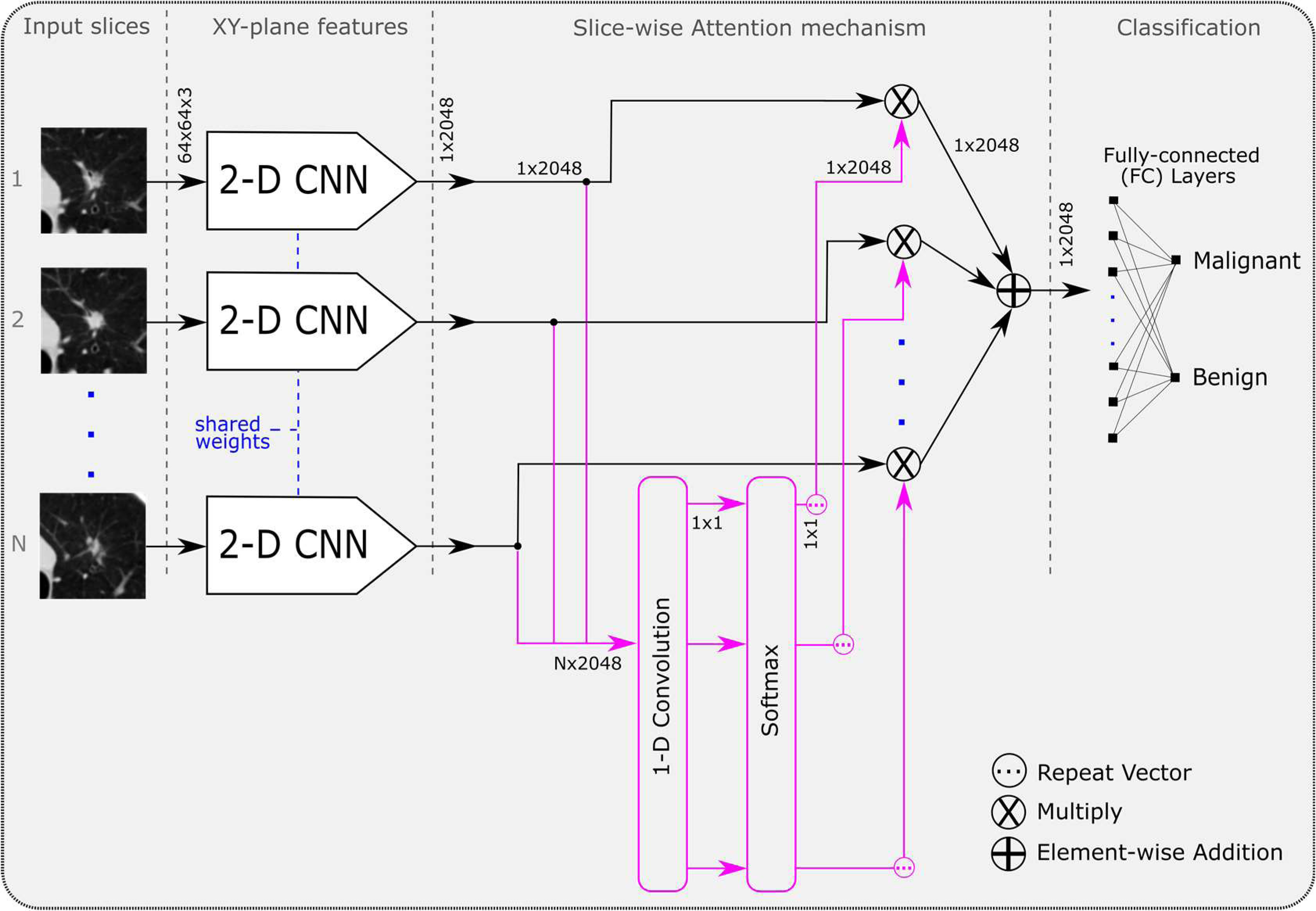
Diagram of the proposed framework for single-time-point nodule classification. A nodule volume (with N=20 slices) is fed to a 2-D CNN slice-by-slice. The slice-wise attention mechanism dynamically weighs the importance of the convolutional features of each slice through learned weights of a 1-D convolutional layer and softmax function. The weighted sum of the attended feature vectors is then used in the final classification layers.

Our proposed framework for single-time-point classification (Fig. 3) uses a 2-D CNN to encode in-plane spatial information while an attention mechanism encodes 3-D information by suppressing irrelevant slices. For this work, we have chosen VGG16 [18] as the feature extractor but any 2-D CNN could be used. The convolutional features for each slice - taken after the final pooling layer - are collectively passed to an attention mechanism adapted from [19] to work with image data that suppresses outputs that are not relevant for the final malignancy classification. The weighted-sum of each 2048-length, 2-D convolutional feature vector – weighted by the corresponding attention value – is then connected to a 128 unit fully-connected layer and final softmax classification layer.

Although training was done using all available nodules from t_0_, t_1_, and t_2_, in order to assess performance on nodules from different time points, testing was done separately on nodules from each screening interval.

The proposed network was trained in one format with and without ImageNet weight initialization, referred to as CAN1-2D and CAN1-R, respectively, but is compared against convolutional recurrent networks (CRN) from our previous work [12] (see Table III for a list of networks used in this paper). Each of these networks were trained using a batch size of 32 samples with the Adam optimizer [20] and 0.0001 learning rate to minimize cross-entropy error.

To measure the efficacy of the proposed approach, the method was tested alongside a 3-D CNN (see Table II). The final classification layers of the 3-D CNN network were chosen to be two fully-connected layers with 128 units each which closely resemble the recurrent module in our previous work or attention mechanism in this paper. Glorot weight initialization [21] was used for this network while the Adam optimizer with a 0.00001 learning rate was used to minimize cross-entropy error.

**TABLE II:** Network Architectures

CAN1-2D	CNN-3D
Input (64x64x3 image)	Input (64x64x20 image)
Conv2D (64, 3x3 kernels)	Conv3D (64, 3x3x3 kernels)
Conv2D (64, 3x3 kernels)	Conv3D (64, 3x3x3 kernels)
Max Pooling (2x2 kernel)	Max Pooling (2x2x2 kernel)
Conv2D (128, 3x3 kernels)	Conv3D (128, 3x3x3 kernels)
Conv2D (128, 3x3 kernels)	Conv3D (128, 3x3x3 kernels)
Max Pooling (2x2 kernel)	Max Pooling (2x2x2 kernel)
Conv2D (256, 3x3 kernels)	Conv3D (256, 3x3x3 kernels)
Conv2D (256, 3x3 kernels)	Conv3D (256, 3x3x3 kernels)
Conv2D (256, 3x3 kernels)	Conv3D (256, 3x3x3 kernels)
Max Pooling (2x2 kernel)	Max Pooling (2x2x2 kernel)
Conv2D (512, 3x3 kernels)	Conv3D (512, 3x3x3 kernels)
Conv2D (512, 3x3 kernels)	Conv3D (512, 3x3x3 kernels)
Conv2D (512, 3x3 kernels)	Conv3D (512, 3x3x3 kernels)
Max Pooling (2x2 kernel)	Max Pooling (2x2x2 kernel)
Conv2D (512, 3x3 kernels)	Conv3D (512, 3x3x3 kernels)
Conv2D (512, 3x3 kernels)	Conv3D (512, 3x3x3 kernels)
Conv2D (512, 3x3 kernels)	Conv3D (512, 3x3x3 kernels)
Max Pooling (2x2 kernel)	Max Pooling (2x2x1 kernel)
Slice-wise Attention	Fully-Connected (128)
Fully-Connected (128)	Fully-Connected (128)
Fully-Connected (2, softmax)	Fully-Connected (2, softmax)

A comparison of the proposed network with its closest 3-D equivalent for single-time-point classification. In the left column, a 2-D network processes incoming 2-D slices from a 3-D scan, and the attention mechanism combines these features into a single feature vector while suppressing irrelevant slice information (see Fig. 3 for more details).

During training, the data were resampled to a uniform resolution and augmented with random flips, shifts, Gaussian noise, and rotations. For the grayscale images to work with 2-D VGG16 networks that operate on RGB images, the CT slices were repeated for the red, green, and blue channels. Each network was trained with stratified 5-fold cross-validation with the same hyperparameters until performance on the validation set plateaued. Here, three folds were used as training, one as a validation, and one as a test set. Each fold included approximately 170 nodules. For training, t_0_, t_1_, and t_2_ images of the nodules were used and were grouped into folds for cross-validation by patient ID numbers to avoid training and testing on nodules of the same patient.

The LIDC data were also used to validate the method. For this data we filtered the scans to include only those with slice thickness ≤ 2.5 mm which is the same as for NLSTx albeit with a slightly lower average thickness. Only nodules that were annotated by 3 radiologists with median malignancy scores < 3 or > 3 were chosen. In total, 647 nodules were used – 387 benign and 260 malignant. The data were similarly resampled to the same resolution as the NLSTx data, and the CNN-3D, CRN, and CAN networks were trained and tested on this data using the same training parameters as for NLSTx.

#### 2) Slice-wise Attention Mechanism

The attention mechanism is used to dynamically weigh the convolutional features of each 2-D slice of the input volume so that the classifier focuses on the features with the most relevant information for malignancy classification. Let *F* be a matrix of convolutional feature vectors [*f*_1_,*f*_2_,…,*f*_N_], where N is the number of slices in the input volume. The attention weights, *α*, are calculated and applied to *F* by:

\begin{align*}
&M = {\rm{tanh}}\left({{w^T}F + b} \right)\tag{1}\\
&\alpha = softmax\left({{M^T}} \right)\tag{2}\\
&d = F\alpha\tag{3}
\end{align*}

**Fig. 4.**
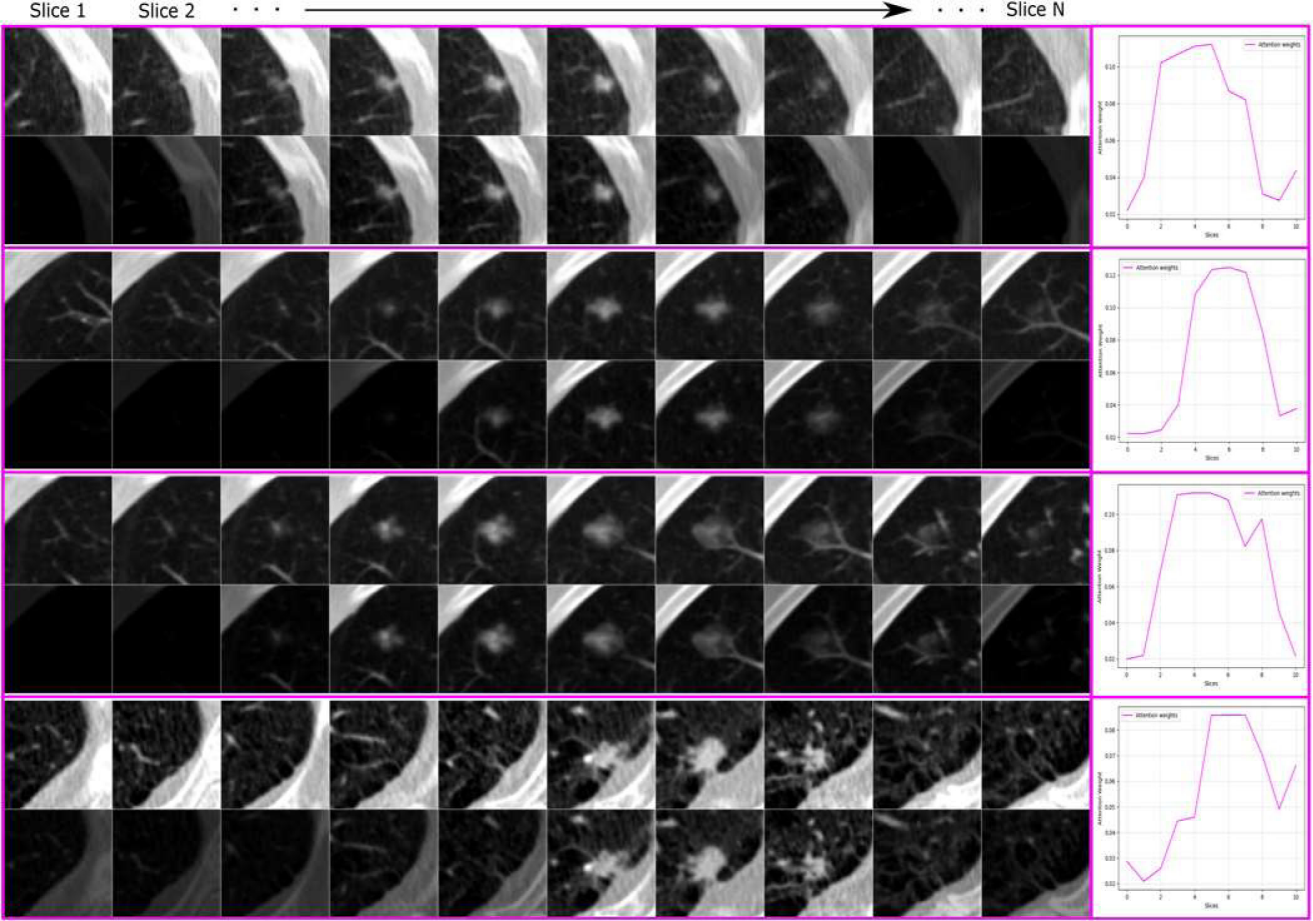
Visualization of attention weighting of the proposed single-time-point network being applied to a set of N (=10) 2-D slices of nodule volumes in NLSTx. To do this, we applied the attention weight (output of the attention mechanism's softmax layer in Fig. 3) to its corresponding input slice. Four nodule volumes are shown with their original slices (top row in each partition) along with their attention-weighted slices (bottom row in each partition). Slices where a nodule is present are brighter (weighted more heavily) than slices where no nodules exist. To the right of each nodule, a plot of the attention weights vs. slices is shown.

Where *d* represents the attention weighted features. Equation (1) shows a single unit 1-D convolutional layer with kernel length 1, weights *w*, bias *b*, and tanh activation that is used to calculate the *importance* of each convolutional feature vector i.e., where to focus attention. Herein, a weight was calculated for each feature vector in *F* between -1 and 1 with a value of 1 being the most important. In (2), a vector of attention weights is produced by softmax that normalizes the values to between 0 and 1. The attention weights were then applied to *F* via a dot product as in (3). The output, *d*, is seen in Fig. 3 as the vector that is passed to the fully-connected classifier. Figure 4 shows the effect of the attention weights when applied to their corresponding input volumes.

The 1-D convolutional filter activates for incoming features that correlate with its learned weights. In our case, Fig. 4 illustrates that the learned filter reacts strongly for features that represent a nodule. For slices that show the center of a nodule, the filter has a strong activation and corresponding attention weight while it has reduced activations and attention weights for slices with smaller, more difficult to see nodules or slices with no nodule whatsoever. This allows for a reduction of features through an attention-weighted summation to a fixed sized output (regardless of the number of input slices) without loss of valuable information for classifying an input volume.

#### 3) Multi-Time-Point Classification

**Fig. 5.**
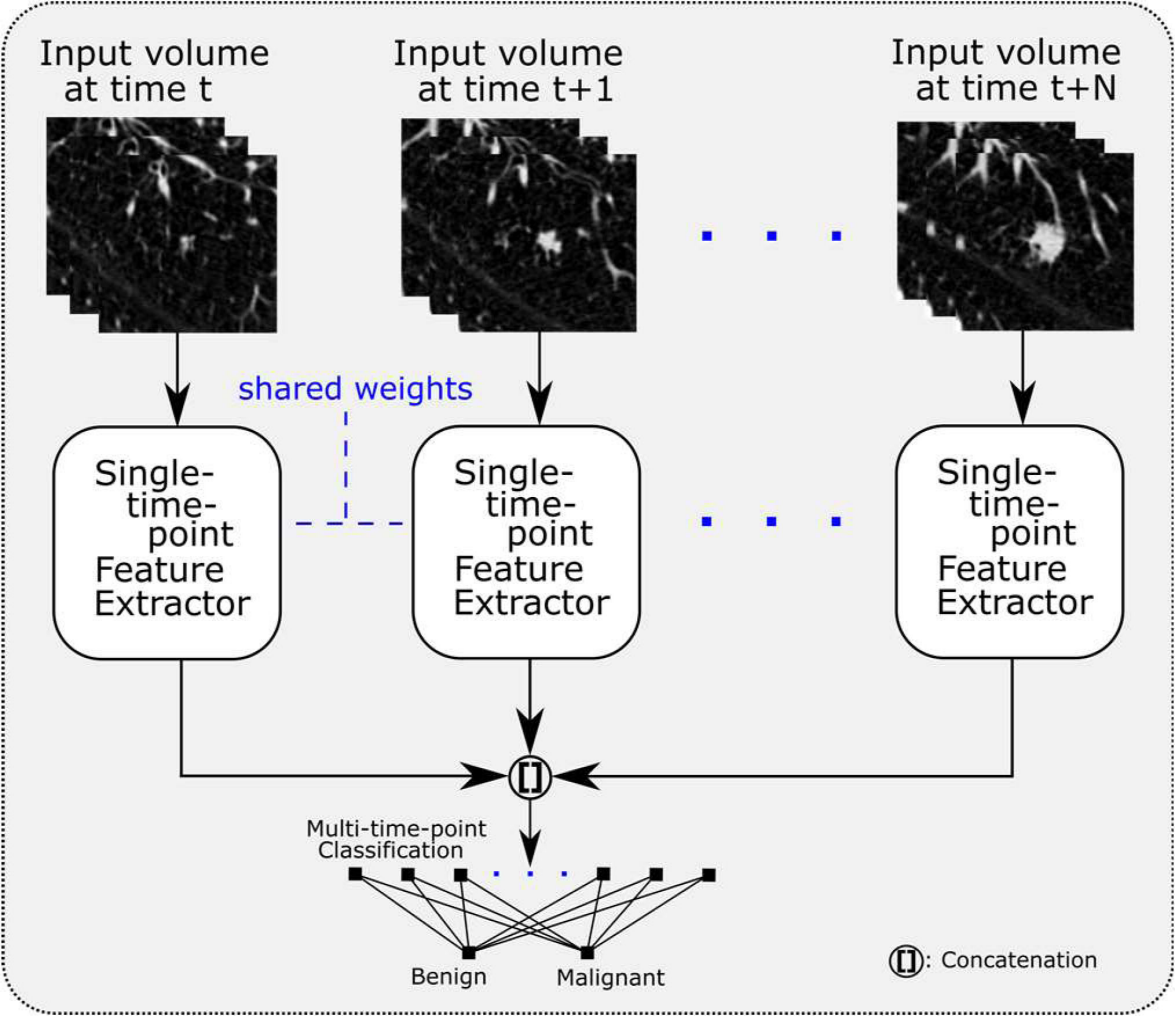
Diagram of the proposed framework for multi-time-point classification through the Siamese scheme. For each input, the same single-time-point network is used as in Fig. 3 (excluding the fully-connected classification layers). The resulting features from each single-time-point feature extractor are concatenated and used in the subsequent fully-connected layers for malignancy classification. This framework is extendible to N time points; we used with two and three time points as available in the NLSTx data.

For multi-time-point classification, each input is processed with the same 2-D CNN and attention mechanism as in single-time-point classification with shared network parameters across inputs (see Fig. 5). The attention-weighted features from each branch are then concatenated before being passed to a 2^(6+M)^ unit fully-connected layer and softmax classification layer where M is the number of volumes in the time series being processed. Conveniently, this approach can be extended to any number of M inputs; the factor of 2^(6+M)^ was empirically found to perform well with a VGG16 backbone CNN. For the NLSTx data, two- and three-branch networks are used, called CAN2-2D and CAN3-2D, respectively.

The preprocessing for multiple time points was identical to single time points except that the data augmentation for input pairs/triplets was done in unison such that each pair/triplet received the same augmentation and remained roughly aligned. The same groupings of patients were used for 5-fold cross-validation as in single-time-point classification. The Adam optimizer was used for training with learning rates of 0.0001 for both the dual- and tri-branch networks.

A description of all networks used in this paper is given in Table III for quick referencing.

**TABLE III:** Deep Networks in This Paper

Network	# of Inputs	Base CNN	Weight Initializations	RNN	Attention
CNN-3D	1	3D VGG16	Random (R)	-	-
CRN1-R	1	VGG16	Random (R)	P	-
CRN1-2D	1	VGG16	ImageNet	P	-
CRN2-2D	2	VGG16	ImageNet	P	-
CRN3-2D	3	VGG16	ImageNet	P	-
CAN1-R	1	VGG16	Random (R)	-	P
CAN1-2D	1	VGG16	ImageNet	-	P
CAN2-2D	2	VGG16	ImageNet	-	P
CAN3-2D	3	VGG16	ImageNet	-	P

Table of networks in this paper and their attributes. CNN-3D is a VGG-16 style 3D CNN. CRNj-2D are convolutional recurrent networks [12] that use recurrent modules to encode slice-wise features. Each CRN network uses different weight initializations or a different number of inputs. CANj-2D are the convolutional attention networks proposed in this paper which are each constructed to process a different number of inputs when considering a time series. In these networks, the index j represents the number of inputs the network accepts. For example, CAN1-R accepts one input while its weights are randomly initialized.

## III. Results

The networks' performances were measured using three metrics: Area Under the Receiver Operating Characteristic Curve (ROC-AUC) [22], Average Precision (AP) [23], and log loss (NLL) [24]. NLL is the negative log-likelihood of the true class given the network's prediction while AP is the approximated area under the Precision-Recall curve; $AP = \ \mathop \sum \nolimits_n ({{R_n} - {R_{n - 1}}}){P_n}$ where ${P_n}$ and ${R_n}$ are the precision and recall at different thresholds, $n$.

While ROC-AUC is a valid metric for comparison of classifiers, other metrics exist that give a different perspective of classifier performance. Therefore, alternative metrics for assessment (i.e., AP and NLL) are reported to help show the proposed method's clinical utility. AP, for instance, helps examine the system's performance in a similar manner to ROC-AUC without considering the number of true negatives (i.e., benign nodules correctly predicted to be benign) in its calculation since true negatives are arguably less clinically significant if mislabeled.

Single-time-point classification was first explored on the NLSTx dataset (see Table IV). For this task, the networks were evaluated on each interval separately as well as all intervals combined. Multi-time-point classification performance is also shown in Table IV when only pairs and the triplet of consecutive intervals are used for training and evaluation, but for these, two- and three-branch networks were used. This allowed classification to be dependent on two or three time points rather than one, like in single-time-point classification.

**TABLE IV:** Single- and Multi-Time-Point Networks on NLSTx

	**Single-time-point NLSTx**												
		Interval t_0_			Interval t_1_			Interval t_2_			All Intervals		
Network	Params	ROC-AUC	AP	NLL	ROC-AUC	AP	NLL	ROC-AUC	AP	NLL	ROC-AUC	AP	NLL
CNN-3D [12]	44.4M	0.798	0.538	0.472	0.815	0.666	0.476	0.841	0.585	0.418	0.817	0.597	0.456
CRN1-R [12]	16.4M	0.737	0.461	0.529	0.765	0.548	0.535	0.774	0.496	0.512	0.759	0.502	0.526
CRN1-2D [12]	16.4M	0.806	0.559	0.396	0.835	0.669	0.407	0.855	0.628	0.337	0.831	0.620	0.381
CAN1-R	14.9M	0.722	0.435	0.580	0.764	0.557	0.568	0.755	0.474	0.565	0.747	0.488	0.571
**CAN1-2D**	**14.9M**	**0.826**	**0.641**	**0.385**	**0.868**	**0.741**	**0.371**	**0.879**	**0.690**	**0.330**	**0.858**	**0.691**	**0.362**
	**Multi-time-point NLSTx**												
		Interval t_0_-t_1_			Interval t_1_-t_2_			Interval t_0_-t_1_-t_2_					
Network	Params	ROC-AUC	AP	NLL	ROC-AUC	AP	NLL	ROC-AUC	AP	NLL			
CRN2-2D [12]	16.4M	0.847	0.684	0.348	0.849	0.659	0.331	-	-	-			
**CAN2-2D**	**15.7M**	**0.879**	**0.773**	**0.305**	**0.884**	**0.757**	**0.322**	-	-	-			
CRN3-2D [12]	16.4M	-	-	-	-	-	-	0.823	0.629	0.396			
**CAN3-2D**	**18.4M**	**-**	**-**	**-**	**-**	**-**	**-**	**0.882**	**0.745**	**0.328**			

Comparison of networks on the NLSTx dataset when tested on single- and multi-time-points (results from proposed networks for single- and multi-time-point classification in bold). Clear improvements in performance are seen by the proposed approach, CAN3-2D, for single-time-point classification when using ImageNet weights and the attention mechanism. Furthermore, the proposed approach uses less than half the network parameters than the closest equivalent 3-D network, CNN-3D. For multi-time-point classification, further gains are seen by using a one- and two-branch attention-based networks with inputs at multiple time points. Please see text for description of metrics.

Single-time-point classification was lastly checked on a well-known dataset, LIDC. The performance measures for the proposed networks are shown beside a 3-D variant in Table V. This LIDC dataset does not contain multi-time-point data; thus, only single-time-point classification is evaluated.

**TABLE V:** Performance of Networks on LIDC

Network	Parameters	ROC-AUC	AP	NLL
Dey et al. [6]	-	0.955	-	-
Kaung et al. [26]	-	0.943	-	-
Safta et al. [27]	-	0.977	-	-
CNN-3D	44.4M	0.914	0.884	0.422
CRN1-R	16.4M	0.900	0.859	0.447
CRN1-2D	16.4M	0.939	0.897	0.317
CAN1-R	14.9M	0.940	0.929	0.305
**CAN1-2D**	**14.9M**	**0.950**	**0.933**	**0.299**

Results for single-time-point classification are shown against competing techniques when separately trained and tested on the LIDC dataset (results from proposed network in bold). We see similar gains in performance with the proposed approach, CAN1-2D, as on the NLSTx dataset.

## IV. Discussion

The proposed method introduces an attention mechanism that weighs 2-D convolutional features of axial slices of a 3-D volume so that they can be reduced by summation to vector of less dimensions. This lowers the overall network parameters and eases training by for the fully-connected classifier. Axial slices were chosen for this work since it is a typical view for diagnosing malignancy; however, this mechanism leaves multi-slice techniques possible, like those in [14].

Owing to the efficiency of the attention mechanism, we can see a noticeable reduction of parameters in the proposed network, CAN1-2D, when compared with CNN-3D and matches that of the convolutional recurrent networks, CRN1-R and CRN1-2D. Instead of using a fully-connected layer that connects to a large feature map as in the 3-D network, the attention mechanism considers smaller feature maps corresponding to each individual slice. This causes a dramatic reduction in the number of parameters of the overall network.

When both the 3-D network, CNN-3D, and the random weight convolutional recurrent network, CRN1-R, are compared, the 3-D network shows superior performance. The power of our proposed framework is introduced by allowing a parameter-efficient way of using pre-trained weight initialization while dramatically reducing the number of network parameters when compared with a 3-D network. This network can match or even outperform the randomly initialized 3-D network on all single-time-point intervals. Since the attention mechanism only contains one learnable kernel, visualization of relevant slices in the input volume that contribute to the final classification is easily determined (as shown in Fig. 4) and helps interpretation of results.

It is clear when comparing the 3-D network and convolutional recurrent networks from our previous work, CRN1-R and CRN1-2D, that ImageNet weights are valuable for increasing classification performances. Since using ImageNet weights that were designed for a 2-D network cannot easily be used in a 3-D network, the slice-wise attention mechanism in this work shows a feasible way to utilize ImageNet weights for a 3-D input. Our proposed single-time-point network with attention, CAN1-2D, outperforms all other networks by a noticeable margin. We believe the attention mechanism eases learning for the fully-connected layer by suppressing irrelevant encodings (as seen in Fig. 4) whereas the recurrent module in CRN1-R and CRN1-2D overwrites and loses important features across slices during encoding. In this way, the classifier receives only the most importance features which allows finer discrimination than when also receiving noisy, irrelevant data.

Comparison with previous studies for single-time-point classification may be made with methods that were validated on biopsied samples. The dataset in [10], [11] contained nodules from the NLST database but had a smaller evaluation set of 237 samples; the reported performances were 0.83 and 0.87 for ROC-AUC, respectively. In a recent paper, authors in [25] validated their method on the entire NLST database (including t_0_, t_1_, and t_2_ data) using the full CT volumes to obtain 0.944 ROC-AUC. While both our dataset and the datasets in these papers used cases from NLST, the specific nodules, patients, and size of the datasets are different. Thus, the performance numbers cited here should only be considered as reference.

Due to lack of biopsy confirmation, one cannot be certain that methods trained on LIDC data are getting trained on data with correct labels and furthermore different publications have used different criteria for labeling malignancy – therefore caution should be exercised when comparing methods on LIDC. Notwithstanding this, in Table V, we compare our network trained on LIDC with previously published methods [6], [27], [28]. Our criteria for nodule selection and ground truth creation for the LIDC data was similar to that in [6]. As maybe gathered from the table, the proposed technique performed well against other methods for single-time-point classification. This is while it used a generic 2-D feature extractor from a pretrained 2-D CNN.

The proposed framework is extendable to take multiple volumes of a time series by using Siamese-like, shared branches. This means that the convolutional branch may be used for both single- and multi-time-point classification by only altering the classification network.

Multi-time-point classification for two time points shows an increase in performance over single-time-point classification for just about every metric with the proposed network, CAN2-2D. This is reasonable because this network can utilize temporal features between two nodules and consistent follow-ups should provide more informative data.

The CRN2-2D and CRN3-2D networks were extended from [12] in this work using the same framework as our proposed network in Fig. 5. As in single-time-point classification, our proposed CAN networks also outperform the CRN networks in multi-time-point classification for similar reasons as stated before.

Interestingly, the three-branch network, CAN3-2D, performs on par with the two-branch network. This is not surprising, however, since most growth/change in nodules occurs rapidly between two time points and less commonly has a slow growth over multiple time points.

## V. Conclusion

This paper made use of a novel dataset of 857 distinct nodules at multiple time points, called NLSTx, using definitive ground truths from the NLST database [12]. When available, we believe it is important for the research community to move toward datasets with definitive ground truth rather than relying solely on radiologists' scores. This will ensure that CADx networks learn appropriate features and are tested on true labels.

The paper proposed a framework for processing the slices of 3-D volumes in longitudinal time series using attention-based 2-D CNNs in a Siamese structure. The framework allows for the use of deep 2-D CNNs that have been optimized on ImageNet, with a significantly lower number of network parameters than a comparable 3-D network. It was observed that with the CAN network initialized with ImageNet weights and a slice-wise attention mechanism to consolidate 2-D convolutional features, performance was significantly higher than an equivalent 3-D CNN trained from scratch. The framework is general and can be used to compare longitudinal data with many time points utilizing the Siamese structure. As expected, the two- and three-branch networks for multi-time-point classification, outperformed single-time-point classification.
